# A Therapeutic Strategy for Spinal Cord Defect: Human Dental Follicle Cells Combined with Aligned PCL/PLGA Electrospun Material

**DOI:** 10.1155/2015/197183

**Published:** 2015-01-28

**Authors:** Xinghan Li, Chao Yang, Lei Li, Jie Xiong, Li Xie, Bo Yang, Mei Yu, Lian Feng, Zongting Jiang, Weihua Guo, Weidong Tian

**Affiliations:** ^1^State Key Laboratory of Oral Diseases, West China Hospital of Stomatology, Sichuan University No. 14, 3rd Section, Ren Min Nan Road, Wuhou District, Chengdu 610041, China; ^2^National Engineering Laboratory for Oral Regenerative Medicine, West China Hospital of Stomatology, Sichuan University No. 14, 3rd Section, Ren Min Nan Road, Wuhou District, Chengdu 610041, China; ^3^Department of Oral and Maxillofacial Surgery, West China School of Stomatology, Sichuan University No. 14, 3rd Section, Ren Min Nan Road, Wuhou District, Chengdu 610041, China; ^4^Department of Pedodontics, West China School of Stomatology, Sichuan University No. 14, 3rd Section, Ren Min Nan Road, Wuhou District, Chengdu 610041, China

## Abstract

Stem cell implantation has been utilized for the repair of spinal cord injury; however, it shows unsatisfactory performance in repairing large scale lesion of an organ. We hypothesized that dental follicle cells (DFCs), which possess multipotential capability, could reconstruct spinal cord defect (SCD) in combination with biomaterials. In the present study, mesenchymal and neurogenic lineage characteristics of human DFCs (hDFCs) were identified. Aligned electrospun PCL/PLGA material (AEM) was fabricated and it would not lead to cytotoxic reaction; furthermore, hDFCs could stretch along the oriented fibers and proliferate efficiently on AEM. Subsequently, hDFCs seeded AEM was transplanted to restore the defect in rat spinal cord. Functional observation was performed but results showed no statistical significance. The following histologic analyses proved that AEM allowed nerve fibers to pass through, and implanted hDFCs could express oligodendrogenic lineage maker Olig2 *in vivo* which was able to contribute to remyelination. Therefore, we concluded that hDFCs can be a candidate resource in neural regeneration. Aligned electrospun fibers can support spinal cord structure and induce cell/tissue polarity. This strategy can be considered as alternative proposals for the SCD regeneration studies.

## 1. Introduction

The infliction of spinal cord injury will bring about a series of severe complications and lead to adverse effects on patients' daily life. The pathological and pathophysiological procedure of spinal cord injury is extraordinarily complicated; besides, axon damage and demyelination are irreversible [1–3]. However the effect of traditional therapeutic methods including pharmacotherapy, surgical intervention, and supportive treatment is often far from satisfactory. In cell therapy for spinal cord injury, various seeding cells such as neural stem cells [4, 5], olfactory ensheathing cells [6], bone marrow derived mesenchymal stem cells [7], and Schwann cells [5] have been adopted. Human dental pulp-derived stem cells and stem cells from human exfoliated deciduous teeth (SHED) [8], as adult dental stem cells, were also explored for their origination from cranial neural crest and expressions of early markers for both mesenchymal and neuroectodermal stem cells [9, 10]. Dental follicle cells (DFCs) were firstly isolated by Wise et al. [11]; they also retain the capability for multipotential differentiation and can be induced to present neurogenesis related behaviors [12–15]. Human DFCs (hDFCs), obtained from wisdom teeth extraction or/and alveolar fossa curettage, have higher proliferation ability than human dental papilla cells which will develop into adults' dental pulp [16]. Different kinds of tooth-derived cells will perform different reactions under particular condition. Compared with SHEDs, hDFCs seemed more inclined to express neurogenic marker MAP2 rather than glial marker GFAP [12]. These findings suggested that hDFCs might be an efficient resource in neural regeneration. Moreover, a considerable dose of tissue containing plenty of hDFCs can be easily obtained via extraction of an unerupted wisdom tooth. Thus, we hypothesized that hDFCs might also serve as a kind of efficient seeding cells in neural regeneration.

As for a sizable spinal cord defect (SCD), a gap or block across the nerve pathway forms, calling for rebridging and reconstruction during therapeutic procedure. Acellular matrix [17], electrospun products [18, 19], self-assembling peptide [18], and many other materials [5, 20, 21] have been reported to act as this “bridge.” They could not only play a role of a “carrier” for growth factors [18] or/and seeding cells [22], but also support the pathway [19].

Based on previous studies, we hypothesized that electrospun biomaterial being seeded with hDFCs can match and repair SCD. This study investigated the effect of hDFCs on electrospun biomaterial and utilized them for the repair of SCD.

## 2. Materials and Methods

### 2.1. hDFCs Culture, Identification, and Neurogenic Induction

hDFCs were isolated and cultured as described previously [14, 23, 24]. hDFCs were obtained from third molars of 18–24-year-old healthy individuals. Dental follicles were aseptically dissected and placed in phosphate buffered solution (PBS). Tissue blocks were incubated in *α*-MEM supplemented with 10% fetal bovine serum (FBS, Hyclone, USA), 100 units/mL penicillin (Hyclone, USA), and 100 mg/mL streptomycin (Hyclone, USA) in a humidified atmosphere at 37°C and 5% CO_2_. After 7 days, hDFCs were observed under a phase-contrast inverted microscope (Nikon, Japan). Cell culture medium was changed every 2 days and cells from passages 2–4 were used for experiments.

2 × 10^4^ hDFCs were seeded on a 6-well culture plate for immunofluorescent staining and neurogenic induction. Neurogenic inducing medium contained 2% dimethyl sulfoxide (DMSO, Solarbio, China), 200 *μ*M butylated hydroxyanisole (Sigma, USA), 25 mM KCl (Kelong, China), 2 mM valproic acid (Sigma, USA), 10 *μ*M forskolin (Sigma, USA), 1 *μ*M hydroxycortisone (Sigma, USA), 5 *μ*g/mL insulin (Gibco, USA), and 2 mM L-glutamine (Sigma, USA) [23]. hDFCs without inducing were used as control group. Samples were fixed with 4% paraformaldehyde in PBS for 30 min. Then the cells were permeabilized by 0.3% triton for 15 min at room temperature. After being rinsed 3 times in PBS, hDFCs were blocked with 1% BSA in PBS (w/v) for 30 min at room temperature in a humidified chamber. After that, they were incubated with primary antibodies overnight at 4°C and washed 3 times in PBS; nuclei were stained with 100 ng/mL of DAPI for 2 min. All samples were examined under a fluorescence microscope (Leica Optical, Germany) and images were analyzed using software Image J (NIH Image, USA). Primary antibodies were anti-vimentin (mouse IgG, 1 : 300, Santa Cruz, USA), anti-CK14 (mouse IgG, 1 : 200, Abcam, UK), anti-nestin (mouse IgG, 1 : 500, Abcam, UK), anti-tubulin *β* III (mouse IgG, 1 : 100, Millipore, USA), and anti-NF200 (mouse IgG, 1 : 200, Abcam, UK). Secondary antibodies were Alexa FluoR 555 goat anti-mouse (1 : 500, Invitrogen, USA).

Flow cytometric analyses were performed to measure expression of mesenchymal stem cell associated surface markers. hDFCs were trypsinized and incubated with CD24 (FITC), CD29 (PE), CD34 (FITC), CD44 (FITC), CD45 (FITC), CD90 (FITC), CD105 (PE), CD146 (PE), and CD166 (PE), which were from BD Biosciences, USA. Flow cytometry was carried out using the Beckman Coulter Cytomics FC 500 MPL system (Beckman Coulter, USA).

For measurement, under the same photographing condition, 4 immunofluorescent samples in each group were picked up randomly and analyzed by Image Pro Plus (Media Cybernetics, USA). Value of integral optimal density (IOD)/nuclear number was graphed by GraphPad Prism 5 (GraphPad Software, USA).

### 2.2. Fabrication and Preparation of Electrospun PCL/PLGA Material

The electrospun PCL/PLGA material was fabricated as previously described [18, 22, 24]. A 3 : 1 mixture of chloroform : methanol by volume was used to create an electrospinning solution of 4.5% poly(*ε*-caprolactone) (PCL, MW 84000, Changchun SinoBiomaterials, China) and 5.5% poly(lactide-co-glycolic acid) (PLGA, 75 : 25, average MW 105000, Changchun SinoBiomaterials, China) by weight. Material was fabricated using an electrospinning machine (Kaiweixin, China) at voltage of 33.0 kV, solution flow rate of 5 mL/h, and distance between spinneret and collector of 20 cm. A roll shaft collector covered with tin foil was utilized to receive aligned electrospun fibers and got aligned electrospun PCL/PLGA material (AEM), as described previously [22]. To create a sleeve structure, AEM was cut into pieces with size of 2 mm × 10 mm along the fibers' direction. The aligned electrospun pieces were soaked in 75% ethanol to remove residual solvent and sterilized for 3 days. After 3 rinses in PBS, these pieces were soaked in alpha-minimum essential medium (*α*-MEM, Hyclone, USA) supplemented with 10% fetal bovine serum (FBS, Hyclone, USA), 100 units/mL penicillin (Hyclone, USA), and 100 mg/mL streptomycin (Hyclone, USA) in a humidified atmosphere at 37°C and 5% CO_2_ until use (at least for 1 day). Scanning electron microscope (SEM, FEI, the Netherlands) was taken to observe surface structure of fibers. Software Image J was used for FFT transformation. FFT transformation images were analyzed by utilizing oval profile of Image J. A circular range (circular center as the image center) was selected and 300 lines at 0–360-degree radius direction of the circle were sampled evenly to calculate the summation of gray value. Then the 300 samples of gray value summation were expressed in spectral intensity distribution.

### 2.3. hDFCs Adhesion on Electrospun Material

SEM visualization of hDFCs adhesion was done by seeding hDFCs on random and aligned electrospun PCL/PLGA material in 24-well culture plates, 1 × 10^4^ cells per well. Samples were incubated for 3 and 5 days, respectively. Fixation was done using 4% glutaraldehyde for 60 min, gradient alcohol series (30, 50, 70, 80, 90, 95, and 100%) for dehydration (15 min each), and hexamethyldisilazane twice for critical point drying. Dried cell-seeded samples were sputtered and examined by scanning electron microscopy.

1 × 10^5^ cells were seeded on a piece of AEM. Cultured under a normal culture condition for 7 days, the hDFCs loaded AEM was fixed for 60 min with 4% paraformaldehyde in PBS, followed by 30% sucrose for dehydration. Freezing microtome (Leica, Germany) was undertaken to get 8 *μ*m thick sections longitudinally for hematoxylin and eosin (HE) staining.

### 2.4. Cytotoxicity and Proliferation

The AEM sample was incubated in *α*-MEM for 3 days at 37°C, while shaking at 100 rpm in an incubator to obtain the extract solution (6 cm^2^/mL). Another *α*-MEM sample incubated for 3 days was used as control. These two kinds of fluid were filtered with 0.22 *μ*m filters. Extract solution was diluted using control sample to the following concentrations: 0% (pure control sample), 25%, 50%, 75%, and 100%. hDFCs, 1000 cells per well, were seeded in 96-well culture plates and then incubated for 4 h at 37°C, 5% CO_2_. Diluted extract solutions were supplemented with 10% fetal bovine serum (FBS, Hyclone, USA), added to the cell-seeded culture plates, and incubated at 37°C, 5% CO_2_. MTT solution (10%, sigma, USA) was added to the wells after 1, 3, and 5 days and reincubated for 3 h.

The AEM sample was cut into small pieces to fit the 96-well culture plates by using a suitable puncher and sterilized before use. The pieces were set into the bottom of the 96-well culture plates; then hDFCs, 1000 cells per well, were seeded in the PCL/PLGA pieces and incubated at 37°C, 5% CO_2_. Cell culture medium was changed every 2 days. hDFCs seeded in the 96-well culture plates immediately were prepared as control. After removing culture medium every day, MTT solution (10%) was added to the wells every day and reincubated for 3 h.

Medium was discarded from the wells and replaced with 150 *μ*L of DMSO to dissolve the formazan salts. Cell viability was quantified by measuring the absorbance at 490 nm using spectrophotometer (Thermo, USA). These experiments were repeated 3 times.

### 2.5. Surgery and Animal Care

A total of 16 adult female Sprague-Dawley rats (250–300 g) were used in this experiment. One day before implantation, hDFCs (1 × 10^6^, GFP-labeled) were seeded on AEM. Experimental rats were anaesthetized using an intraperitoneal injection with 10% hydral (0.3 mL for every 100 g weight). After laminectomy being performed at levels T10 and T11, the left or right side of dura was opened and 2 mm homolateral spinal cord was cut off. A craniocaudal defect of spinal cord was made and allowed accommodation of graft, while a piece of AEM (2 mm × 10 mm, with or without hDFCs) was rolled into a spiral shape cylinder, to fit the lesion. Then wound was covered by using a piece of superficial fascia and closed in layers. Experimental rats were randomly divided into four groups for functional recovery testing: normal control group (laminectomy only, *n* = 3), SCD group (no transplantation after SCD, *n* = 3), AEM group (aligned electrospun PCL/PLGA material transplantation after SCD, *n* = 5), and AEM-hDFCs group (hDFCs + aligned electrospun PCL/PLGA material transplantation after SCD, *n* = 5). After receiving surgery, rats were given extensive care.

### 2.6. Behavior Observation

One day postoperatively and weekly thereafter, experimental rats were functionally monitored and tested using the Basso-Beattie-Bresnahan (BBB) locomotor rating scale. Two examiners who were blinded to the animal's treatments performed the tests [8, 17].

### 2.7. Histology and Immunofluorescence

8 weeks postoperatively, the rats were anaesthetized and cardiac perfusion was taken by using 0.9% physiological saline, followed by 4% paraformaldehyde in PBS. The spinal cord specimens were obtained and dehydrated in 30% sucrose. Sections (8 *μ*m thick) were cut longitudinally and transversely by using freezing microtome for HE staining and immunofluorescent staining.

Sections were stained with hematoxylin and eosin according to the manufacturer's recommended protocol. For immunofluorescent staining, sections were incubated with primary antibodies: anti-GFP antibody (rabbit IgG, 1 : 200, Zen, China), anti-GFP antibody (mouse IgG, 1 : 500, Tianjin, China), anti-hypophosphorylated neurofilament H antibody (NF200, mouse IgG, 1 : 200, Abcam, UK), anti-GFAP antibody (rabbit IgG, 1 : 300, Abcam, UK), and anti-Olig2 antibody (rabbit IgG, 1 : 200, Abcam, UK). PBS was used for negative controls instead of the primary antibodies. For fluorescence, secondary antibodies were Alexa FluoR 555 goat anti-mouse (1 : 500, Invitrogen, USA), Alexa FluoR 488 goat anti-rabbit (1 : 500, Invitrogen, USA).

### 2.8. Statistical Analysis

All measurements, including cytotoxicity, proliferation, and fluorescent optical density, were collected from 4–6 samples and subjected to a paired Student's *t*-test. All data were expressed as the mean ± standard deviation. Statistical significance was analyzed using IBM SPSS Statistics 21.0 software (SPSS, USA). Analysis of variance followed by the nonparametric test of multiple independent samples was used to assess the significant differences of multiple samples and a value of *P* < 0.05 is considered statistically significant.

## 3. Results

### 3.1. hDFCs Culture, Identification, and Neurogenic Induction

**Figure 1:**
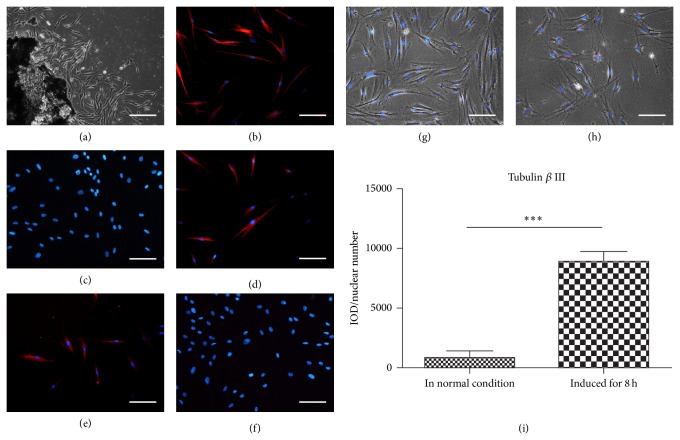
hDFCs culture, identification, and neurogenic induction. (a) hDFCs isolated and cultured for 7 days. (b–f) Immunofluorescent staining for hDFCs: (b) positive for vimentin, (c) negative for CK14, (d) positive for nestin, (e) positive for tubulin *β* III, and (f) negative for NF200; (g) hDFCs stained for tubulin *β* III; (h) hDFCs underwent neurogenic induction for 8 h, presented morphologic change, and were stained for tubulin *β* III; (g) and (h) were observed under the same photographing condition; (i) comparison of the fluorescence of tubulin *β* III for hDFCs between normal condition and 8 h induced condition. IOD/nuclear number was used for measurement. ^***^ *P* < 0.0001. Scale bar = 100 *μ*m.

hDFCs were observed crawling out from the tissue blocks incessantly in the first week (Figure 1(a)). hDFCs from passages 2–4 were characterized by immunofluorescent staining. The spindle shaped cells were strongly positive for vimentin, marker for mesenchymal stem cells, and negative for CK14, an epithelial cell marker, and they were also stained positively for some neurogenic lineage markers such as nestin (neural stem cell marker) and tubulin *β* III (early neuronal cell marker), but negative for NF200, a mature neuronal cell marker (Figures 1(b)–1(f)).

**Figure 2:**
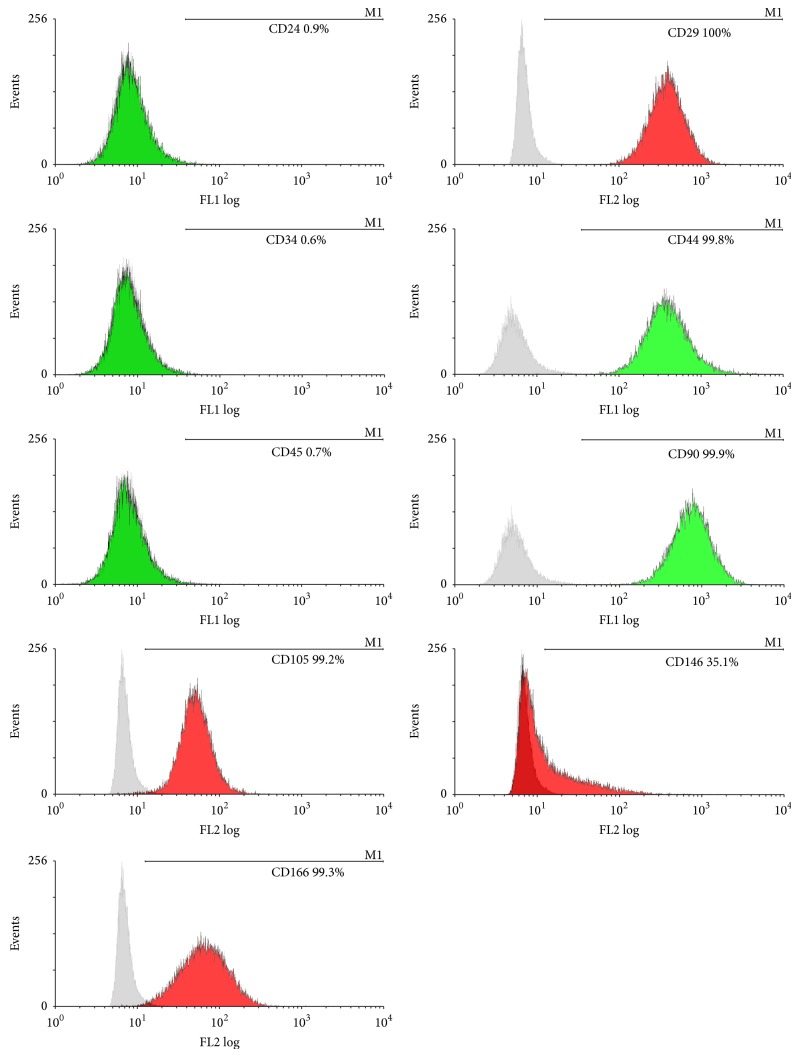
Flow cytometric analyses for immunophenotypic characteristics of hDFCs. hDFCs were positive for CD29, CD44, CD90, CD105, CD146, and CD166 and negative for CD24, CD34, and CD45. Green color stands for being labeled by FITC and red color stands for being labeled by PE.

Flow cytometric analyses showed that hDFCs were positive for CD29 and CD105 (adhesion molecules), CD146 and CD166 (mesenchymal stem cell markers), CD44 (receptor molecule), and CD90 (extracellular matrix protein). But CD24, CD34, and CD45 (haematopoietic and angiogenic lineage markers) were negative (Figure 2).

After being cultured in neurogenic inducing medium for 8 h, the hDFCs underwent morphological changes (Figure 1(h)). Notably, the fluorescence for tubulin *β* III was enhanced obviously. Images taken randomly were analyzed by Image Pro Plus, and result showed that fluorescence for tubulin *β* III was significantly stronger than control (without being induced) (Figures 1(g)–1(i), *P* < 0.0001).

### 3.2. Electrospinning PCL/PLGA Material and hDFCs Adhesion

**Figure 3:**
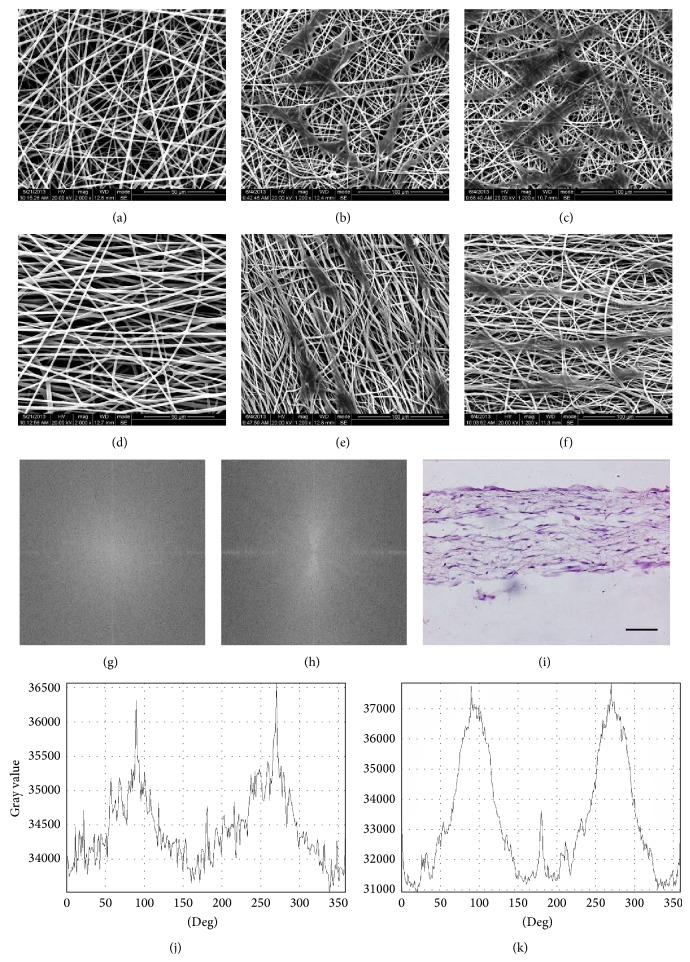
Comparison of random electrospun PCL/PLGA material and aligned electrospun PCL/PLGA material. (a, d) SEM for random (a) and aligned (d) electrospun PCL/PLGA material. FFT transformation was performed for random (g) and aligned (h) fibers. There was obvious diversity of their directional properties. (b, c, e, f) hDFCs seeded on the electrospun material: (b, e) for 3 days, (c, f) for 5 days, (b, c) for being seeded on random electrospun PCL/PLGA material, and (e, f) for being seeded on aligned electrospun PCL/PLGA material. hDFCs could stretch along the direction of fibers as time went on. (i) HE stained for the sections of hDFCs seeded aligned electrospun PCL/PLGA material. (j, k) Spectral intensity distribution at 0–360-degree radius direction verified the diversity between random electrospun PCL/PLGA material (j) and aligned electrospun PCL/PLGA material (k). Intensity was expressed by summating gray value at 0–360-degree radius direction on a circular range of FFT transformation image (circular center coincided with image center). (j) Random electrospun PCL/PLGA material and (k) aligned electrospun PCL/PLGA material. Scale bar = 100 *μ*m.

SEM and FFT transformed image showed the fibers collected by roll shaft had a clearer directional property than the random fibers collected by a plate (Figures 3(a), 3(d), 3(g), 3(h), 3(j), and 3(k)). In the FFT transformation image, gray value distribution of aligned electrospun PCL/PLGA material (Figure 3(h)) was more regular than that of random electrospun PCL/PLGA material (Figure 3(g)). In spectral intensity distribution, gray value summation of aligned electrospun PCL/PLGA material ranged from 31000 to more than 37000; it was wider than that of random electrospun PCL/PLGA material which ranged from 34500 to just above 36500. And the distribution curve of aligned electrospun PCL/PLGA material was smoother (Figures 3(j) and 3(k)). It verified that fibers of aligned electrospun PCL/PLGA material were more oriented. Fibers' diameter was 1403 ± 378 nm (aligned) and 949 ± 377 nm (random).

On the 3rd day when hDFCs seeded, it seemed that hDFCs were likely to orient along the fibers' direction. On random fibers, hDFCs stretched randomly; and on aligned fibers, hDFCs roughly followed the mainstream (Figures 3(b) and 3(e)). On the samples which were cultured for 5 days, we observed length of hDFCs was increased along the mainstream of oriented fibers (Figure 3(f)).

HE staining also showed hDFCs seeded on AEM could stretch along the oriented fibers (Figure 3(i)).

### 3.3. Cytotoxicity of AEM and Proliferation of hDFCs on AEM

**Figure 4:**
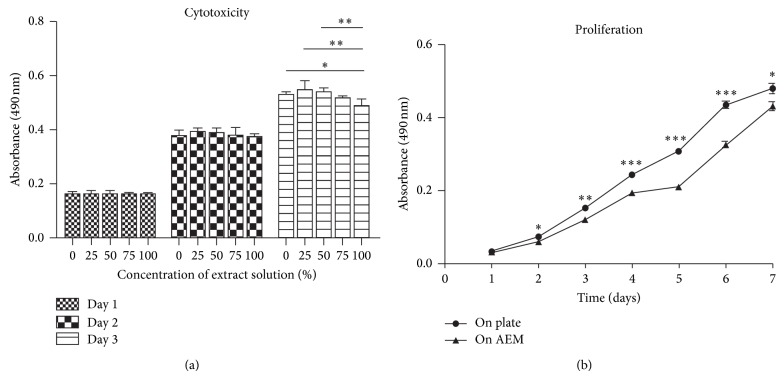
Cytotoxicity of AEM and proliferation of hDFCs on AEM. MTT assays showed that electrospun PCL/PLGA would not induce a cytotoxicity reaction (a) and can support hDFCs proliferating (b). ^*^ *P* < 0.05, ^**^ *P* < 0.01, and ^***^ *P* < 0.0001.

Cytotoxicity was determined using diluted extract solutions of AEM samples. After one-day culture for hDFCs, there was no diversity among all groups in absorbance. As time went on, diversity began to present. On the 5th day, the extract solution group (100%) could still support around 80% of cell viability (Figure 4(a)).

MTT assays were also used to evaluate proliferation of hDFCs (Figure 4(b)). Growth curve of hDFCs was observed for 7 days. Proliferation capacity of hDFCs which were cultured on AEM was a little weaker than those which were seeded on culture plate directly, especially on the 4th to 6th day (*P* < 0.0001, extremely significant). But after that, straight slope at the 6th to 7th day was 0.82 ± 0.09, while on plate it was 0.34 ± 0.10 and the difference was statistically significant (*P* < 0.01); that is to say, hDFCs on AEM showed a fast increasing trend. On the 7th day, the diversity between 2 groups became narrowed. It seemed that the growth curve of 2 groups might have a tendency of confluence in next days.

### 3.4. Functional Recovery Observation

Surgeries were performed on 16 rats, and BBB rating scale was used to assess the locomotor functional recovery of hind limb. Rats in normal control group did not perform locomotor dysfunction and got 21 points. And functional recovery occurred in all individuals in the other 3 groups except one individual in AEM group after 4 weeks postoperatively. At the 8th week, they could get 5–12 points (except one in AEM group still getting 0 points), but there was no significant difference among the 3 groups (data not shown).

### 3.5. Histology and Immunofluorescence

**Figure 5:**
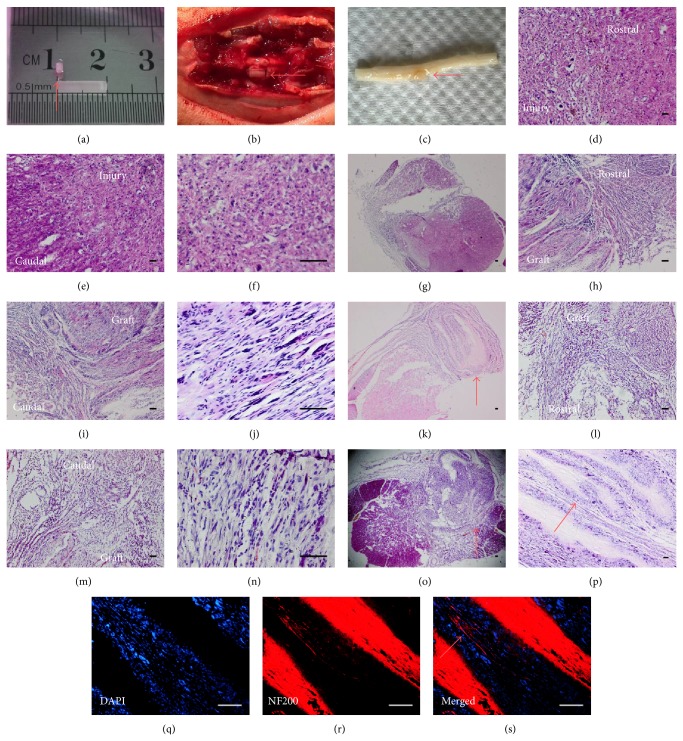
Implantation of graft and histologic analysis. (a) A 2 mm × 10 mm piece of aligned electrospun PCL/PLGA material was rolled and used for implantation; red arrow (a) showed the direction of its mainstream fibers. The graft just fitted the defect (b) and, after 8 weeks, the graft was coupled in the spinal cord (c); red arrows (b, c) indicate the graft and the direction of mainstream fibers. HE staining was undertaken in SCD group (d–g), AEM-hDFCs group (h–k), and AEM group (l–o). In longitudinal sections (d–f, h–j, l–n), especially in the injured site (f) and in the graft (j, n), it is shown that cellular direction in graft is obviously clearer than that in injured site without graft being transplanted. (g, k, o) Transverse section was also stained for its structure observation; red arrow (k, o) showed the graft. (p–s) A stream of fibers (red arrows in (p) and (s)) passed through the AEM and immunofluorescent staining showed these fibers were positive for NF200 (q–s). Scale bar = 100 *μ*m.

**Figure 6:**
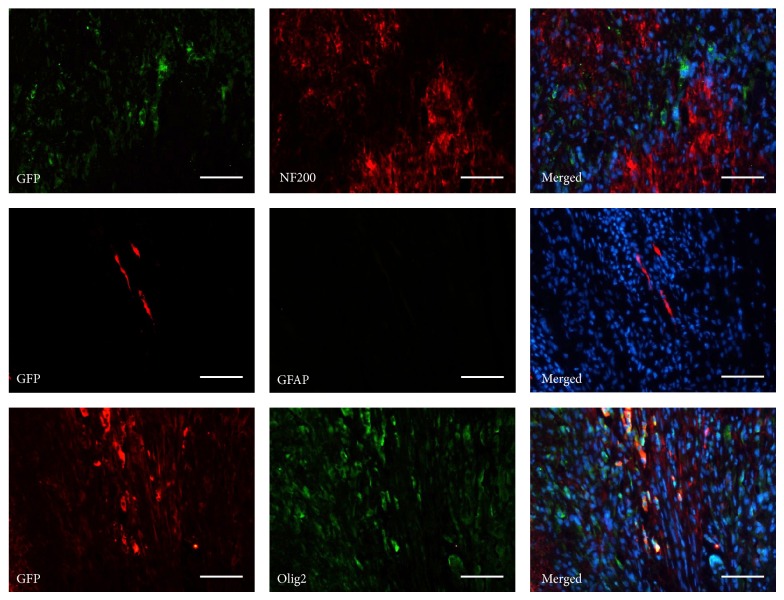
Immunofluorescent analysis for neurogenic characteristics of implanted hDFCs. GFP-labeled hDFCs were negative for NF200 and GFAP, but they could be positive for Olig2. Scale bar = 100 *μ*m.

The specimens and HE staining showed that 8 weeks postoperatively, the graft had been tightly coupled to the spinal cord and took place of the lesion (Figures 5(c), 5(h)–5(k), and 5(l)–5(o)). In AEM-hDFCs group and AEM group, a great quantity of cells migrated into AEM and the spindle shaped cells could follow the orientation of AEM (Figures 5(j) and 5(n)). GFP (+) cells were detected in AEM-hDFCs group; they expressed neither NF200 nor GFAP but were positive for Olig2 (Figure 6). But there were no GFP (+), Olig2 (+) cells detected in AEM group. In SCD group, a amorphous structure or a cavity took the place (Figures 5(d)–5(g)). A more interesting finding was that a stream of fibers which were detected in AEM group reached AEM (Figure 5(p)). Under immunofluorescent staining, these fibers presented positive for NF200 (Figures 5(q)–5(s)).

## 4. Discussion

Tissue engineering techniques have been widely studied aiming at curing spinal cord injury and have got significant achievements currently. Since multipotential capability of tooth-derived cells have been proved [8–10, 12–15], they were also utilized for tissue engineering attempts. A very important reason they were frequently studied by so many researchers is that they could be obtained without any adverse influences on human body. Unlike neural stem cells, application of tooth-derived cells will not raise ethical questions. hDFCs, which could easily propagate to a great quantity, possess huge potential in regenerative medicine. Although cell therapy has always been a hot topic in these decades, single-handed application has its limitations. When a certain scale lesion or a particular structure deficiency has been inflicted, a piece of cell mass can hardly reconstruct appropriate morphology or function well. That is why biomaterial scaffold should be added to rebuild certain structures. PLGA, poly(L-lactide) (PLLA), gelatin, and collagen were attempted for nerve reconstruction as electrospun materials [18, 19, 25]. Since PCL-based materials could maintain a long term mechanical strength [26] and the longitudinal aligned electrospun fibers were clearly superior to randomly aligned fibers in neural regeneration [22, 27], longitudinal aligned PCL/PLGA electrospun material was selected for connecting the gap in spinal cord [18]. Overall, we tried to combine cell therapy and biomaterial, specifically, which was hDFCs implantation combined with longitudinal aligned electrospun PCL/PLGA material.

Owing to the fact that hDFCs expressed both mesenchymal and some neurogenic markers, it was supposed that hDFCs would contribute to neural regeneration. Previous experiments indicated that DFCs could upregulate the expression of neurogenic markers such as nestin, tubulin *β* III, NSE, and neurofilament under a certain condition [13, 15]. Here, our study* in vitro* also noticed that, besides morphological changes occurring in hDFCs, tubulin *β* III was highly expressed after a neurogenic inducing procedure. These neurogenesis related behaviors suggested that hDFCs might be of promising prospect in SCD treatment.

Aligned electrospun PCL/PLGA material was fabricated as scaffold for spinal cord reconstruction and carrier for hDFCs. An increasing PLGA concentration could increase cellular attachment, proliferation, and spreading [28]; hence, the PCL/PLGA material in this study was fabricated at the proportion of 4.5 : 5.5, a high proportion of PLGA. To test cytotoxicity of AEM and proliferation of hDFCs on AEM, MTT assays were performed. In our study, incubated medium was utilized to dilute the extracted solution and to be the control sample. Instead of utilizing fresh medium, this modification could exclude the changes which came from the difference between fresh medium and incubated medium and hold a better homogeneity. Compared with previous study [29], our results indicated that, besides the fact that no cytotoxic effect was induced, AEM could support an excellent growth curve. So we confirmed that AEM is a kind of desirable biomaterial in tissue engineering uses.

After hDFCs being seeded overnight on AEM, hDFCs had deposited certain amount of extracellular matrix and adhered tightly to AEM. It made the implantation procedure simpler: there was no need to prepare cell suspension. Thus this strategy can be prospective in operability. Moreover, this strategy can be more promising if drug(s) loaded or nerve growth factor(s) encapsulated electrospun material was achieved [30, 31].

Trying to verify what roles hDFCs and AEM played from perspective of praxeology, functional recovery observation of 4 experimental groups was taken for 8 weeks postoperatively. After anesthesia, rats in normal control group woke up without lower limbs dysfunction. It testified that animals which received laminectomy only did not have spinal cord injury. Most individuals in the other 3 groups showed functional recovery more or less after 4 weeks postoperatively, but it failed to be significant statistically. We considered the reasons insufficient samples should be the main cause. In this study, we set 16 rats, a relatively small sample size, for behavior observation. So, individual difference would make a relatively large influence on the result. Secondly, although researchers were proficient to operate SCD model, the boundary of lateral spinal cord defect was not so easy to control as that of spinal cord transverse defect. Preservation of contralateral function would unavoidably increase the difficulty of consistency maintenance. But, on the other hand, spinal cord transverse defect was inevitable to give rise to much higher mortality, even with high level of postoperative nursing. In a word, different SCD model has its advantages and disadvantages and researchers should make a decision on the contradiction in consideration of particular circumstances. In this study, probably a further enlargement of sample size can make a statistically significant result from praxeological perspective.

Although we failed to achieve a positive result from praxeological observation, in histological analyses we got some interesting findings. The graft had integrated in the defect area of spinal cord and connected both sides of the lesion after 8 weeks postoperatively in AEM group and AEM-hDFCs group. And under high-magnificent image we noticed a large number of aligned cells in the graft. In transverse section, the graft was observed to take the place of lesion site. On the contrary, a big cavity formed and blocked the continuity of spinal cord in SCD group. From simple morphological observation, it seemed that there were no obvious differences between AEM group and AEM-hDFCs group; in other words, AEM with or without hDFCs being seeded could not only support the structure of spinal cord but also hold a good biocompatibility and play a role of vectoring cell polarity. Since implanted hDFCs were detected to express GFP immunofluorescence in the graft, we thought of the fact that survived hDFCs played a role of homing [32] at target site. At the same time, the survivals were able to express Olig2, an oligodendrocyte precursor cell marker, but NF200 and GFAP were negative in these cells. This result was similar to previous study in SHEDs [8]. Although no neurogenic differentiation was observed, this oligodendrogenic lineage differentiation of implanted hDFCs could substitute dead oligodendrocytes and benefit remyelination in central nervous system, furthermore, to promote functional recovery [33]. As we know, reactive astrogliosis which can be stained GFAP (+) will inhibit reconnection of axons and be obstacles in nerve pathway [34]. Rather than reactive astrogliosis, the implanted hDFCs seemed more inclined to perform “oligodendrogliosis” in SCD microenvironment. That is to say, rather than limiting nerve growth, implanted hDFCs were more inclined to benefit remyelination. In this study, another significant finding was that NF200 (+) fibers were detected to pass through AEM. It suggested that implanted AEM provided a scaffold to permit nerve fibers to reach into and it was potential to rebuild the blocked nerve pathway. Since AEM allowed nerve fibers to pass through and hDFCs could do a favor in remyelination, we inferred that hDFCs and AEM could contribute to SCD treatment.

In conclusion, hDFCs can hold neuroregenerative abilities and be utilized as a practical cellular resource for the treatment of SCD. Implanted hDFCs can survive and tend to differentiate towards oligodendrogenic lineage in the microenvironment of spinal cord defect. Aligned electrospun fibers can not only support spinal cord structure but also induce cell polarity and permit nerve fibers to pass through. This strategy can be considered alternative proposals for the SCD regeneration studies.

## Abbreviations

SCD: Spinal cord defect
DFCs: Dental follicle cells
hDFCs: Human dental follicle cells
AEM: Aligned electrospun PCL/PLGA material
SHED: Atem cells from human exfoliated deciduous teeth
IOD: Integral optimal density
PCL: Poly(*ε*-caprolactone)
PLGA: Poly(lactide-co-glycolic acid)
AEM-hDFCs: Aligned electrospun PCL/PLGA material with human dental follicle cells being seeded
BBB rating scale: Basso-Beattie-Bresnahan rating scale.
